# Atypical age-related changes in cortical thickness in autism spectrum disorder

**DOI:** 10.1038/s41598-020-67507-3

**Published:** 2020-07-06

**Authors:** Adonay S. Nunes, Vasily A. Vakorin, Nataliia Kozhemiako, Nicholas Peatfield, Urs Ribary, Sam M. Doesburg

**Affiliations:** 1 Department of Biomedical Physiology and Kinesiology, Simon Fraser University, 8888 University Dr, Burnaby, BC V5A 1S6 Canada; 2 Behavioral & Cognitive Neuroscience Institute, Simon Fraser University, Burnaby, Canada; 3 Department Pediatrics and Psychiatry, University of British Columbia, Vancouver, Canada; 4 B.C. Children’s Hospital Research Institute, Vancouver, Canada; 5 Department Psychology, Simon Fraser University, Burnaby, Canada

**Keywords:** Development of the nervous system, Autism spectrum disorders

## Abstract

Recent longitudinal neuroimaging and neurophysiological studies have shown that tracking relative age-related changes in neural signals, rather than a static snapshot of a neural measure, could offer higher sensitivity for discriminating typically developing (TD) individuals from those with autism spectrum disorder (ASD). It is not clear, however, which aspects of age-related changes (trajectories) would be optimal for identifying atypical brain development in ASD. Using a large cross-sectional data set (Autism Brain Imaging Data Exchange [ABIDE] repository; releases I and II), we aimed to explore age-related changes in cortical thickness (CT) in TD and ASD populations (age range 6–30 years old). Cortical thickness was estimated from T1-weighted MRI images at three scales of spatial coarseness (three parcellations with different numbers of regions of interest). For each parcellation, three polynomial models of age-related changes in CT were tested. Specifically, to characterize alterations in CT trajectories, we compared the linear slope, curvature, and aberrancy of CT trajectories across experimental groups, which was estimated using linear, quadratic, and cubic polynomial models, respectively. Also, we explored associations between age-related changes with ASD symptomatology quantified as the Autism Diagnostic Observation Schedule (ADOS) scores. While no overall group differences in cortical thickness were observed across the entire age range, ASD and TD populations were different in terms of age-related changes, which were located primarily in frontal and tempo-parietal areas. These atypical age-related changes were also associated with ADOS scores in the ASD group and used to predict ASD from TD development. These results indicate that the curvature is the most reliable feature for localizing brain areas developmentally atypical in ASD with a more pronounced effect with symptomatology and is the most sensitive in predicting ASD development.

## Introduction

Autism Spectrum Disorder (ASD) is a highly heterogeneous neurodevelopmental disorder that has an early-life onset. It is characterized by deficits in social communication and restricted or repetitive behaviors and/or interests^1^. Several neuroimaging studies have reported region-specific changes in brain morphology in ASD^2–4^ supported by post-mortem studies^5^. Findings of atypical brain morphology in ASD, however, are highly heterogeneous^6–8^, and there are not yet clear neuroanatomical markers for accurately identifying individuals with ASD.

Recent studies have used developmental changes to identify individuals with ASD. For example, Bezgin et al.^9^ used longitudinal changes in cortical white–gray contrast to predict ASD diagnosis and severity using the ABIDE repository; Emmerson et al.^10^ obtained functional connectivity features from 6-month-old children to predict ASD diagnosis at 24 months of age. Similarly, Bosl et al.^11^ used EEG data at several age periods from 3 to 36 months to identify ASD. Previously, brain morphometric measures such as cortical thickness (CT) and cortical volume have been used to predict ASD^12–15^, and this remains a promising feature to classify ASD based on developmental changes.

One challenge to classify ASD is the heterogeneous nature of the disorder^16–18^, which, together with small sample sizes and varying age ranges in some studies, it might partially explain inconsistencies in the neuroimaging literature. One hypothesis is that such inconsistencies are due to the age-specific developmental atypicality of the ASD brain. These developmental trajectories were studied either through modeling age-related changes characterizing cross-sectional samples of participants^9,19^ or exploring longitudinal changes in neuroimaging or neurophysiological markers^11^. Specifically, a number of studies have recently supported this, reporting atypical developmental trajectories in the ASD brain in terms of neuroanatomy^20–22^, hemodynamic functional connectivity^23–26^ and neurophysiological rhythms and synchrony^19,27,28^.

Significant alterations of developmental trajectories of CT have been reported in ASD. For example, it has been shown that in ASD adults CT decreases more dramatically with age compared to typically developing (TD) adults^29^. Increased CT in children with ASD has also been reported in children aged 8–12 years^3^. Zielinski et al. reported three distinct phases of atypical cortical development in ASD: an accelerated expansion during early childhood, accelerated thinning in later childhood and adolescence, and decelerated thinning in early adulthood^30^. Wallace and colleagues, conversely, reported accelerated age-related cortical thinning in temporal and parietal areas during early adulthood in the ASD population^31^. In addition, based on a sample of 6–15 year old children with ASD, Jiao and colleagues found both increased (in the precuneus and anterior cingulate cortex) and decreased (in the frontal pole and parahippocampal gyrus) thickening^12^. Recently, Khundrakpam et al.^31^ using the ABIDE I repository, reported higher CT in frontoparietal areas in ASD until adolescence, where accelerated thinning was found until adulthood.

In the present study, we investigated group differences and prediction accuracies in terms of various polynomial models, namely, linear, quadratic, and cubic models, for age-related changes in CT estimated at three different levels of spatial coarseness, using three parcellations with different number of regions of interest (ROI). Specifically, for each model, we estimated its highest order coefficient. These constant share parameters characterize the entire sample used to estimate them, and have a geometric interpretation of the slope, curvature, and aberrancy for the linear, quadratic, and cubic polynomial models of age-related changes, respectively. In our context, the linear slope represents a constant change in CT over age, with the curvature representing an acceleration in the rate of changes in the slope and the aberrancy is defined as the rate at which cortical thickness's acceleration changes with respect to age. In our study, we refer to these first, second, and third derivatives of the corresponding linear, quadratic, and cubic polynomial models as the shape parameters of the developmental trajectories of cortical thickness.

For each MRI scan, CT was estimated at three levels of spatial coarseness: hemispheric parcellation (HP) with two trivial ROIs (left and right hemispheres), FreeSurfer Anatomical Parcellation (FSAP) using the Desikan-Killiany atlas^32^ with 68 brain areas, and Multi-Modal Parcellation^33^ (MMP) with 360 brain areas. Our aim was to test if CT, estimated using these three parcellations, was atypical in ASD, and to explore to what degree the age-related changes in CT a sensitive measure to differentiate ASD and TD populations. After mapping atypical trajectory shapes in different areas of the cortex, we studied their relationship with ASD symptomatology by associating them with ADOS scores. Then, with machine learning (support vector machine), we assessed if the linear slope, curvature, and aberrancy of the age-related trajectories could be used to classify ASD and TD development.

## Results

### No significant group differences in CT across a wide age range

**Figure 1:**
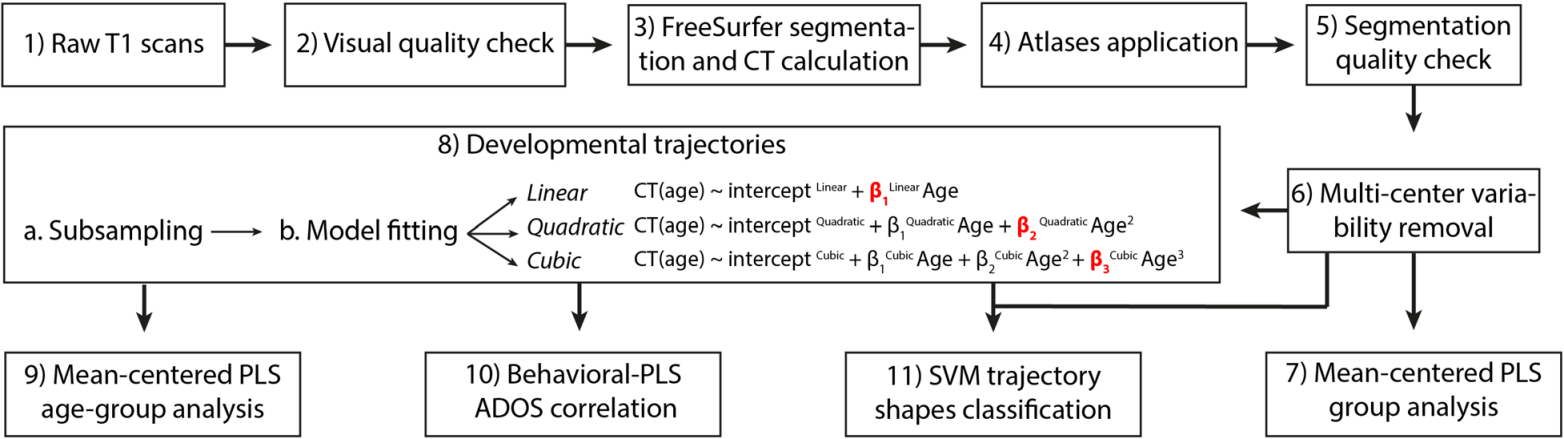
The workflow of the analyses. The coefficients in red in step 8 are the trajectory shapes used in the subsampling, correlation, and classification analyses. β_1_^Linear^ is the coefficient describing the slope, β_2_^Quadratic^ the curvature, and β_3_^Cubic^ the aberrancy.

After a series of pre-processing steps (see Fig. 1, steps 2–6, and Methods for more detail), we tested for overall group differences in CT, separately for the HP, FSAP, and MMP parcellations. We applied multivariate analysis called mean-centered Partial Least Squares (PLS), taking into account all the features of a parcellation (CT estimates for different ROIs) at once (see “Methods” and Fig. 1—step 7). One “global” permutation-based test was applied to evaluate the statistical significance of overall group differences (one p-value for all the features), and a bootstrap test was performed to quantify the reliability of a feature contribution to the group contrast. The result of the bootstrap test is a vector of z-scores, each associated with the ROIs. The larger-in-modulus z-scores indicate the most reliable effects. They can be positive or negative and are defined with respect to the hypothesis “CT for ASD is higher than CT for TD”. The PLS analysis revealed no significant group differences (p-values > 0.05) in CT between ASD and TD groups for all the parcellations.

### Atypical developmental trajectories of CT in ASD

**Figure 2:**
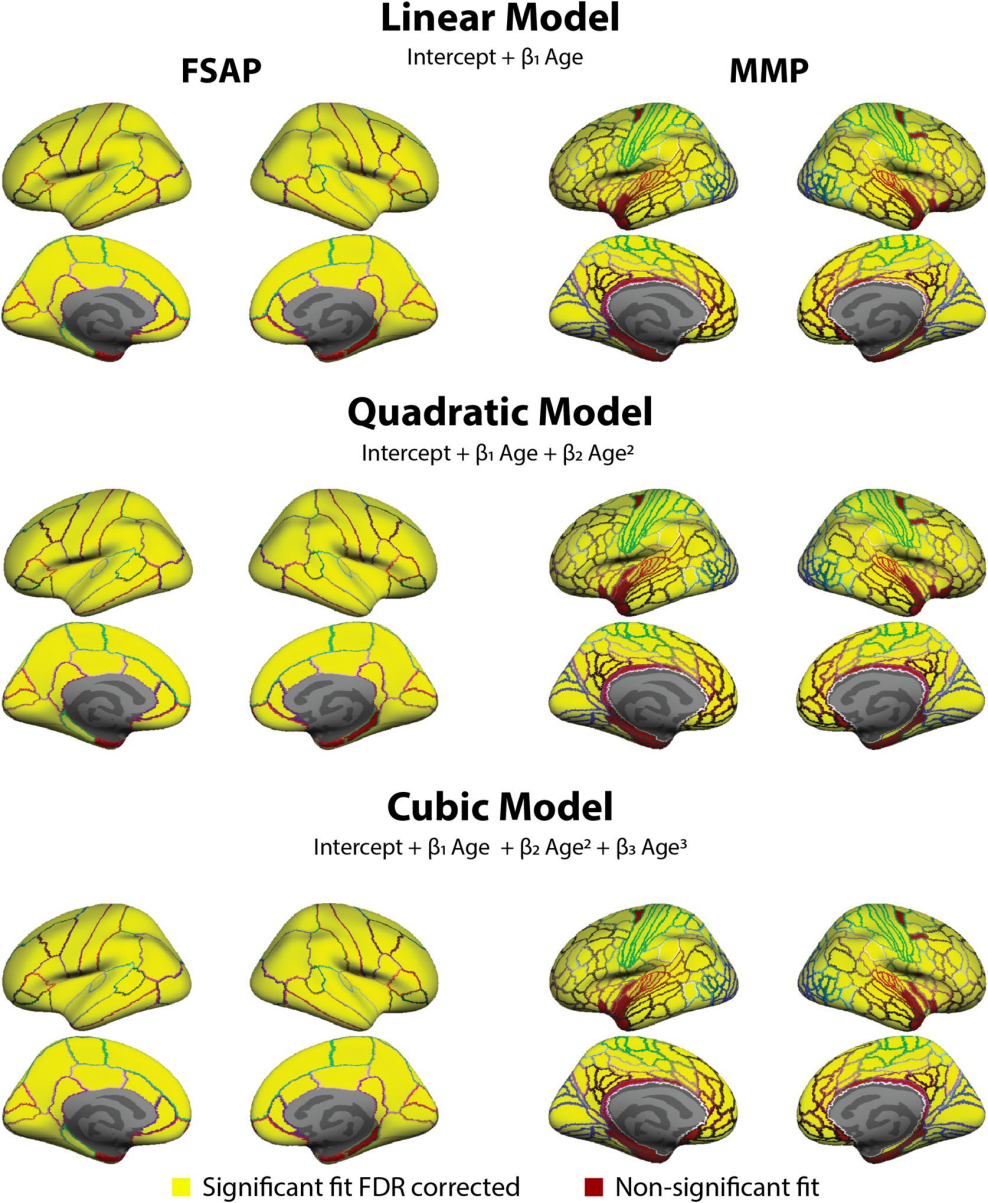
Areas from the Free Surfer Anatomical Parcellation (FSAP) and Multi Modal Parcellation (MMP) used in the analyses. The goodness of fit at each area of the FSAP and MMP were measured with a deviance test assessing if the model significantly fits better than a constant model. After FDR correction, areas with a significant fit with an FDR corrected p-value < 0.05 were used in subsequent analyses (colored in yellow), and non-significant were masked out (colored in red). The model fit was done separately for each group and deemed non-significant if it was not in any of the groups. Both areas of the HP significantly fit and are not plotted. Colored areal border outlines represent the original atlas annotation color of the FSAP (left) and MMP (right).

Having found no significant group differences in CT across the entire age range, we explored group differences in terms of the shape parameters of the developmental trajectories. Specifically, a linear, quadratic, and cubic polynomial model was fitted separately for each group and parcellation to characterize age-related changes in CT for each brain area. First, we used the entire sample (separately for each group) to test the goodness of fit for each ROI with a deviance test. Figure 2 illustrates the model fit for the three models and three parcellations. A few brain areas around the ventral medial temporal cortex did not significantly fit, after FDR correction, in some of the models and especially with the MMP. These areas were masked out and excluded from further analyses.

The slope, curvature, and aberrancy of the corresponding linear, quadratic, and cubic polynomial models are contract parameters, by design. They characterize the entire sample used to estimate them. To test for group differences, we needed to have multiple estimates of them. To introduce a variability in the model coefficients, we generated multiple sub-groups of participants as random samples without replacement, subsequently fitting each of the three models to each sub-group. Specifically, we generated 80 sub-groups composed of 70 subjects. For each ROI in all the three parcellations, we estimated the slope, curvature, and aberrancy of the age-related changes in CT. Further, these estimates were statistically assessed for group differences with the mean-centered PLS, performed separately for each parcellation. The estimates of the slope, curvature, and aberrancy were tested with PLS simultaneously, but separately for each parcellation.

**Figure 3:**
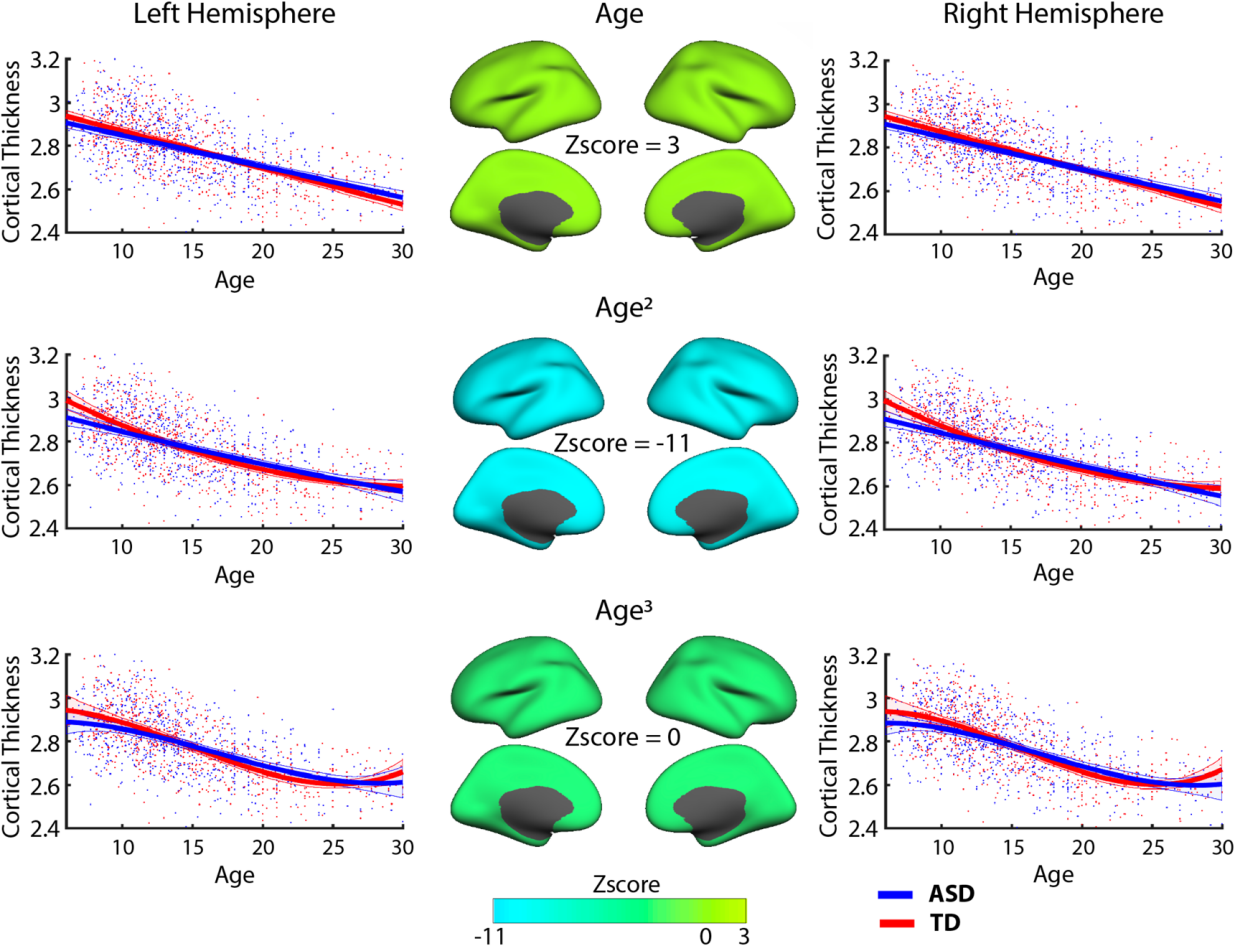
Developmental trajectories of CT for the hemispheric parcellation. The linear slope, curvature, and aberrancy from the three models (linear-top; quadratic-middle; cubic-bottom row) were estimated to quantify differences in age-related changes (trajectories) between ASD and TD. In the middle column, z-scores from the PLS analysis are plotted for the right and left hemisphere representing how reliable the model coefficients were in expressing the overall group differences. The trajectory shape represented by the curvature coefficient was most reliable, followed by the slope. In the trajectory sub-plots, red and blue lines represent the CT trajectories averaged across sub-groups, with the shade areas limited by their one standard deviation on each side. The points represent the individuals. CT measures are measured in millimeters (after removing the inter-center variability).

We found statistically significant differences in the shape of age-related trajectories in CT for all three parcellations, HP (p < 0.01), FSAP (p < 0.001), and MMP (p < 0.001), after Bonferroni correction. Specifically, for the HP parcellation, PLS indicated strong effects for the slope and curvature. Figure 3 illustrates the average hemispheric trajectories (across sub-groups) and the z-scores distribution: negative for the curvature, and positive for the linear slope. Z-scores are the largest in modulus for the curvature, followed by the slope, indicating the more reliable effects. The aberrancy has z-scores close to zero, implying that it is not a feature sensitive for group differences. In Fig. 3 (hemispheric parcellation), we observe a negative linear slope for both groups, but the slope is steeper for TD. In other words, ASD is characterized by a reduced rate of cortical thinning. At the same time, the trajectory for TD has a positive curvature. It is a convex curve, wherein a straight line joining any two points on the curve lies totally above the curve. For ASD, the curve is almost flat, with the curvature value close to zero. As illustrated in Fig. 3, the ASD developmental trajectory for the quadratic model at earlier ages has a decreased rate of thinning compared to TD development and at early adulthood ASD CT become similar to the TD group.

**Figure 4:**
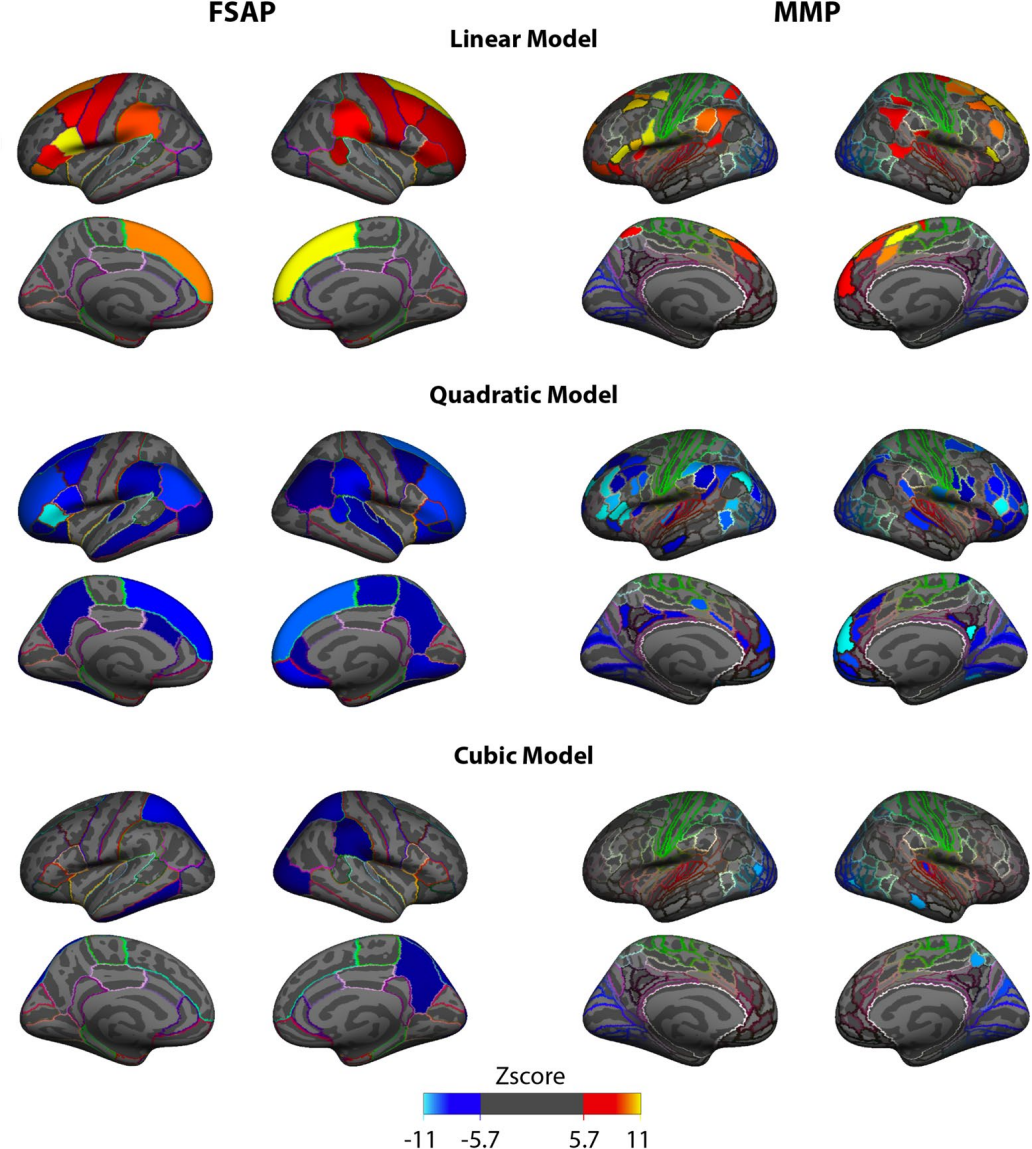
Distributions of the z-scores across brain areas, shown separately for the three polynomial models, where the highest order feature is taken, and two atlases (left for FSAP, right for MMP). For the group contrast (ASD > TD) the main effect for the slope and the curvature is represented by the positive and negative z-scores, respectively. The spatial distribution of the largest z-scores for the slope is very similar to the spatial distribution of the z-scores for the curvature from the quadratic models. The colors of the outlines in each cortical area correspond to the colored area of the atlas.

For the FSAP and MMP, PLS analyses rendered results similar to the HP. Specifically, the distributions of z-scores for the slope and curvature were spatially similar but with opposite sign z-scores. Figure 4 shows the distribution of z-scores for all the three shape parameters, separately for the FSAP and MMP. Z-scores are to be interpreted with respect to the hypothesis ASD > TD: increases in a shape parameter in ASD are associated with positive z-scores, and vice versa. Brain areas with the highest in modulus z-scores representing the most reliable group differences were located in the frontal, fontal-medial and temporoparietal junction (TPJ) areas for the linear and quadratic models. In addition, for the quadratic model and MMP parcellation, high z-scores were localized in the posterior cingulate cortex (PCC), and in the FSAP, the precuneus. In contrast, largest z-scores for the aberrancy were located in occipital and posterior-parietal areas.

**Figure 5:**
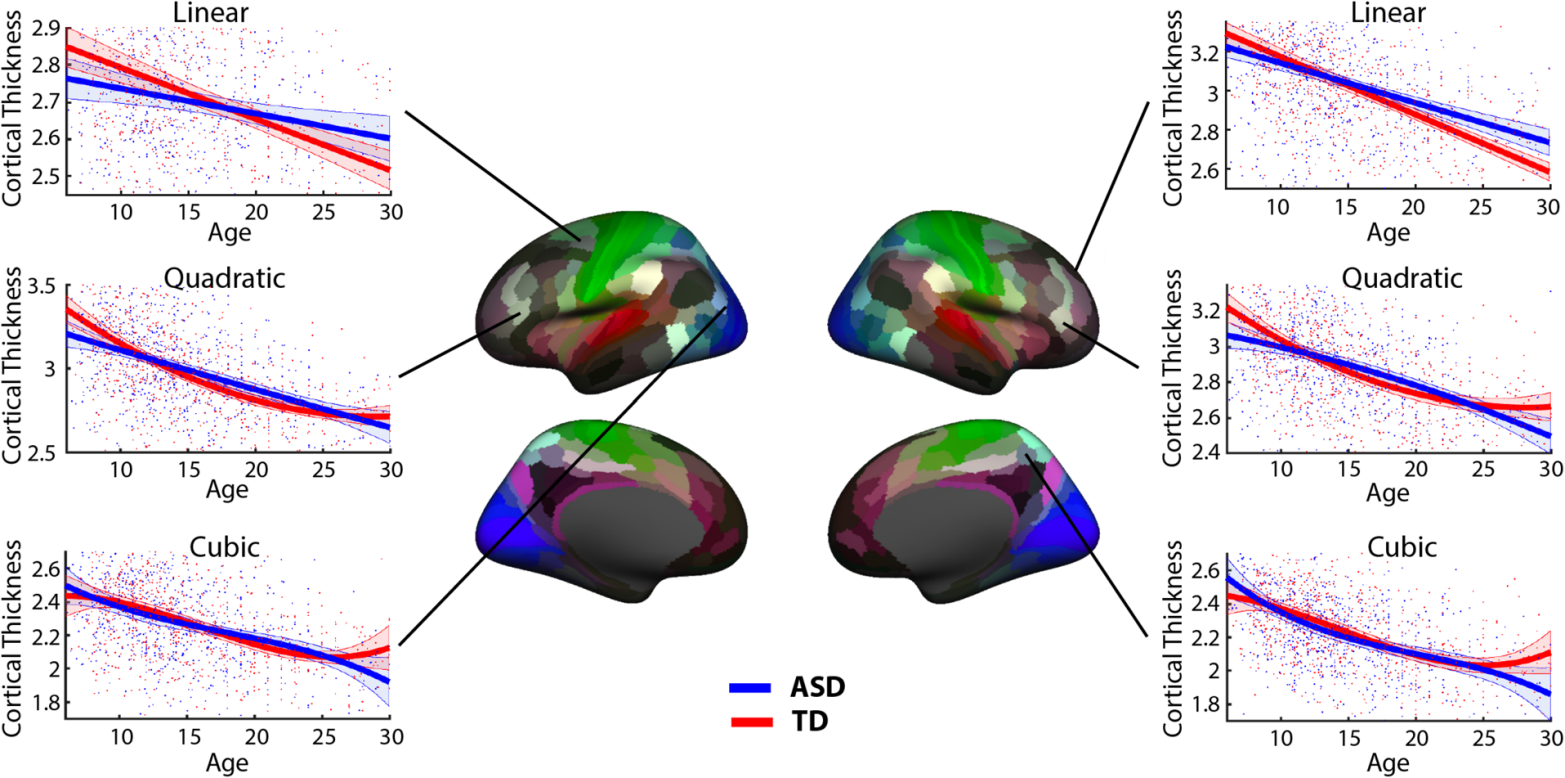
Developmental trajectories of cortical thickness (CT) for the brain areas from the MMP atlas, which reliably expressed group differences. Trajectories are plotted, separately for each model, for a few areas with high z-scores in the right and left hemisphere. For a given ROI, red and blue lines represent the CT trajectories averaged across sub-groups, with the shade areas limited by their one standard deviation on each side. The points represent the individuals. CT measures are measured in millimeters (after removing the inter-center variability).

Similarly, as in the HP analysis, the ASD development was characterized by decreased cortical thinning. Specifically, the slopes were negative for both groups, but more positive in the ASD, implying a reduced rate of thinning in ASD (slower decline). Most of the areas with largest negative z-scores for the curvature coefficients were negative in the ASD group, while in the TD were positive. Supplementary Fig. S1 plots the areas of the MMP with negative curvatures and they considerably overlap with the areas with the most significant z-scores of the PLS analysis for the curvature. Figure 5 illustrates linear, quadratic and cubic age-related trajectories for three ROIs at each hemisphere with high z-scores. Although a negative curvature could imply both an increase and decrease in the rate of thinning, in our age range we captured a reduction in CT between childhood and early adulthood, as noted in the trajectory plots in Figs. 3 and 5. Thus, we interpret negative curvatures in the context of CT decreasing with age, and they indicate an initial decreased rate of thinning at earlier age and increased thinning with age, whereas the slope captures a constant rate of thinning. To find the point in the quadratic model, where negative CT curvatures in ASD transitions from slowing to increased thinning, FSAP and MMP areas with negative curvature values in the ASD subgroups were selected. Then a straight line was created between the CT values at 6 and 30 years old and the furthest point in the negative curvature from the straight line was calculated. The most distant to the straight line, the turning point, was localized on average at 18 years of age.

### One-center analysis of developmental trajectories of CT in ASD

To further support our findings based on the analysis wherein batch effects (multi-center variability) had to be removed, we repeated the same analysis using participants from only one center, when the potentially confounding effects of multi-center variability are not present. We observed similar results for the slope with positive z-scores, and for the curvature with negative z-scores. Given the spatial overlap with the PLS analysis with all the centers, the similarity suggests that the effects of constant decreased thinning captured by a less steep slope and an increasing thinning captured by a more negative curvature, were found within the NYU dataset. As can be seen in Supplementary Fig. S2, areas with the highest z-scores for the linear and quadratic highest order coefficients are also located in frontal and temporal lobes, TPJ and PCC. The spatial distribution of the z-scores for the cubic coefficient did not spatially correspond, suggesting once more that the aberrancy of the cubic model did not capture reliable group effects.

### Associations between ADOS and developmental trajectories of CT

To explore the relationship between ASD symptomatology and the shape parameters of the CT trajectories of the three models, we performed a series of multi-variate analyses (separately for each parcellation and model coefficient). Specifically, we applied Behavioral PLS analysis (see methods for more details) to explore correlations between two multi-variate data sets organized as matrices: (i) the linear slope, curvature, or aberrancy of the age-related trajectories estimated for individual sub-groups, and (ii) ADOS-Generic scores (communication, social and stereotyped behavior subscales) averaged across the participants composing the given sub-groups. As in the group analysis, wherein we had to introduce a variability of the shape parameters through sub-sampling of the entire data, we used the same approach here to generate a certain number of sub-groups of subjects. To account for possible different distributions of z-scores across ROIs, we performed six different behavioral-PLS analyses (three shape parameters times two parcellations, FSAP and MMP), and the p-values obtained for each model were Bonferroni corrected.

**Figure 6:**
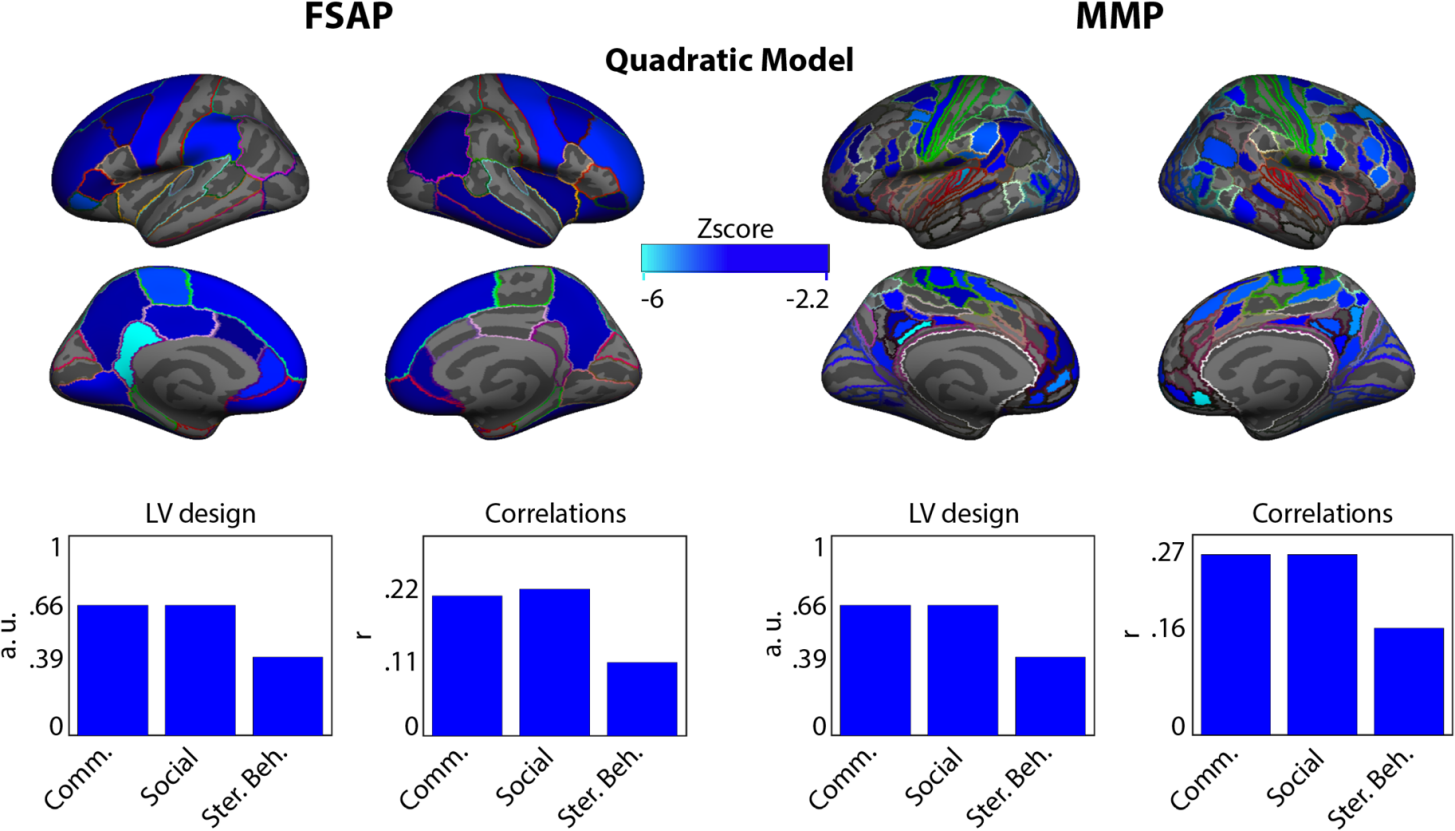
Overall correlations between the curvature values of the age-related trajectories of cortical thickness (CT) in the ASD group and the mean ADOS scores averaged across the participants to characterize the curvature using the quadratic model. The spatial map in blue indicates negative z-scores representing the association between higher ADOS scores and smaller values of the estimated curvature coefficient of the quadratic developmental trajectories (curvature itself could be positive or negative). On the bottom left, the latent variable (LV) design illustrates the bar plots for each atlas indicating the contribution of the ADOS scores to the z-scores (how much each contributed to the overall correlation). On the bottom right, are indicated the overall ADOS scores correlations with all the curvature coefficients.

We found that only the overall correlations between the curvature and the ADOS variables were significant for both FSAP and MMP parcellations: p < 0.01 after Bonferroni correction. Their distributions of z-scores were skewed towards negative values. PLS z-scores should be interpreted with regard to the LV design variable (a group contrast, as in the analysis of group differences, or overall associations between the curvature and ADOS, in this case). Together with all positive values of the LV design variable, a distribution of z-scores skewed towards negative values indicates overall negative correlations between the curvature and ADOS scores. The more negative the curvature, the more severe the symptomatology. More specifically, the overall correlations between the curvature coefficients from the FSAP and ADOS sub-scores were: r = − 0.21 for communication subscale, r = − 0.22 for social subscale and r = − 0.11 for stereotyped behavior subscale. For the MMP, the correlations were: r = − 0.27 for communication subscale, r = − 0.27 for social subscale and r = − 0.16 for stereotyped behavior subscale. Figure 6 illustrates the latent variable (LV) design reflecting the contribution of each ADOS sub-score, their correlation values with curvature coefficients, and the spatial distribution of the most negative z-scores. The spatial distribution of the most negative z-scores was very similar to that of the group differences analysis, being mostly located in frontal areas, TPJ and PCC.

### ASD classification of CT developmental trajectories

We tested to what degree the shape parameters can predict if a given trajectory shape represents ASD or TD changes in CT. Specifically, to support the discriminatory power of the shape parameters for developmental prediction, a classification analysis using a support vector machine was applied to predict developmental trajectories of ASD versus TD populations based on either their slope, curvature, or aberrancy. We applied this analysis to the FSAP and MMP parcellations. Table 1 reports the total accuracy, specificity, and sensitivity means computed across 500 cross-validation runs, and Table 2 illustrates if the mean of the classification metrics between models of the FSAP or MMP were significantly different.

**Table 1:** Classification metrics using different model coefficients with the MMP and FSAP.

	Accuracy			Sensitivity			Specificity		
	Mean	Std	CI	Mean	Std	CI	Mean	Std	CI
**MMP**									
Linear	72.96	11.7	53–91	65.18	25.23	16–97	80.74	20.59	36–100
Quadratic	75.76	10.42	57–91	73.21	22.91	29–100	78.31	20.79	36–100
Cubic	66.51	12.04	47–84	62.6	25.94	14–96	70.43	23.03	26–99
**FSAP**									
Linear	70.62	11.88	51–87	65	25.86	16–97	76.24	21.47	30–100
Quadratic	74.07	10.03	57–89	71.5	22.81	27–99	76.64	20.1	40–99
Cubic	61.57	12.15	41–81	60.33	26.01	13–97	62.82	24.61	17–96

The table illustrates the mean, standard deviation (std) and the 5 and 95th confidence intervals (CI) for the accuracy, sensitivity and specificity using an SVM to classify ASD and TD trajectory features of CT represented by the highest order coefficient of a linear, quadratic and cubic models using areas from the MMP and FSAP.

**Table 2:** Significant differences between models’ classification.

	Accuracy		Sensitivity		Specificity	
FSAP	Linear	Quadratic	Linear	Quadratic	Linear	Quadratic
Quadratic	↓**		↓**		x	
Cubic	↑**	↑**	↑*	↑**	↑**	↑**

MMP	Linear	Quadratic	Linear	Quadratic	Linear	Quadratic
Quadratic	↓**		↓**		↑*	
Cubic	↑**	↑**	↑**	↑**	↑**	↑**

An independent t-test was used to test models’ differences in the mean cross-validation accuracy, sensitivity and specificity presented in Table 1. p-values were corrected for multiple comparisons using FDR. (**) represents a p-value < 0.01 and (*) a p-value < 0.05, FDA corrected non-significant contrasts are represented with an (X). Arrows ↑ and ↓ indicate if the mean of the model in the column is significantly higher or lower than the mean of the model in the row.

Similar to the group analysis, the best total accuracy in classifying ASD and TD was achieved using the quadratic trajectory shape with an accuracy of 76% with the MMP and 74% with the FSAP, followed by the linear (73% and 71%) and cubic (67% and 62%). Highest sensitivity in detecting ASD was achieved with the curvature parameters from the MMP (73%), whereas highest specificity in detecting TD trajectories was achieved with the slope parameter from the MMP (80%). When testing if mean subgroup ADOS values or the number of females per subgroup were correlated with accuracy, sensitivity or specificity, no significant correlations (p-values > 0.05) were found.

## Discussion

In this study, we investigated atypical developmental trajectories of cortical thickness (CT) in the ASD from 6 to 30 years of age. Previously, age-related changes in CT were studied by fitting a linear, quadratic and cubic models^28–31,34^. Instead, in this study, we focused on the highest order derivative coefficient (the slope, curvature and aberrancy) to obtain an age-constant parameter that has an interpretable geometrical shape. These “trajectory features” describe a shape of the developmental trajectory of CT that pertains to the entire age range of the ASD and TD groups. We explored these features at several levels of spatial coarseness using a hemispheric HP, anatomical FSAP and multi-modal MMP atlases. Overall, our results indicate that ASD trajectory features are atypical in ASD in frontal, parietal and midline areas and correlated with ASD symptomatology. In addition, the curvature coefficient of the quadratic model was the most accurate and sensitive in discriminating TD and ASD development, highlighting the importance of this geometrical shape in characterizing ASD atypical development.

Reliable group differences in the shape of CT trajectories were found using the quadratic and linear models for age-related changes in CT. Skewed z-score distributions indicated that, on average, the curvature is more negative, whereas the linear slope was less negative in ASD. The typical development of CT was characterized on average by a negative slope with constant CT thinning over time using a linear model and by a U-shaped positive curvature reflecting a non-linear thinning of CT. In the ASD, curvature values were slightly less negative in the hemispheric parcellations, denoting overall cortex-wise decreased thinning, and on other parcellation areas with large z-scores were mostly negative. Our results showed a skewed distribution, indicating that this developmental process is altered in ASD. The slope and curvature coefficients although having opposite signs, represented a similar effect and the spatial representation of the strongest z-score values greatly overlapped. The trajectory shape captured by the quadratic model significantly correlated with the subgroup-averaged ADOS scores, indicating the greater the symptomatology of the subsample the more negative was the curvature coefficient. Areas with the most reliable correlation of curvature coefficients and ADOS scores tended also to be the most reliable in reflecting group differences in curvature values between ASD and TD. This supports the association between higher ASD symptomatology and a more negative CT developmental curvature.

Our results are consistent with other studies which revealed increased CT in ASD individuals during childhood and early adolescence^3^, reflecting a smaller decrease of CT in this period for individuals with ASD. A study with a large sample size of 3,000 participants, found that alterations in the CT are more pronounced during adolescence, exhibiting an accelerated rate of cortical thinning in frontal areas and decreased in temporal areas^35^. A recent CT study using the ABIDE I also found increased CT in the ASD from early childhood to adolescence and equal or reduced CT in early adulthood in frontal and parietal areas, indicating decreased CT thinning during childhood and adolescence and accelerated thinning in late adolescence^34^. Studies using an age-range similar to ours concluded that the cortex thins less in ASD compared to typically developing controls^3,36^. In addition, the developmental trajectories of the TD group of our study are very similar to the ones described in the longitudinal study of Zielinski et al., however, our ASD trajectories differ with that study with respect to the period of accelerated thinning, namely starting at a later age than in the population studied by Zielinski and colleagues.

The areas with the highest z-scores from the slope and curvature coefficient analyses included areas of the default mode network (DMN) and the frontoparietal network (FPN). Several ASD studies have found alterations in the DMN and FPN involving atypical functional connectivity patterns^26,37–39^, more variability in the spatial networks^16,17^, as well as altered gyrification and structural network architecture in ASD^21,40^. The DMN has been linked to theory of mind, social cognition and inner referential processes^41–46^. One of the characteristics of the DMN is to be deactivated during an externally gated task^47–49^, and DMN tends to be anticorrelated with the FPN which is a goal oriented network^46,50,51^. It has been reported that less DMN deactivation is associated with more stressful performance during stimulus-driven tasks^52,53^, and it has been found that in ASD task deactivation of the DMN is reduced, which is understood to be related to sensory feedback feedforward alterations^54–56^. Moreover, typical development of the DMN displays a sparse pattern of mostly local connections early in development, moving towards a more long-range and cohesive network throughout maturation^57,58^. Also, there is evidence that long-distance connections fail to develop during adolescence in ASD population^59^. Our study is consistent with the aforementioned literature indicating alterations in areas of the DMN and some of the FPN and it suggests that typical cortical neural development is altered and might underpin alterations of structure–function relations in areas of the DMN and FPN.

The trajectory shape for CT in the TD group represents a neurotypical U-shaped thinning centered around the age of pubescence and early adulthood^30,60^, whereas in the ASD group, there is a decreased thinning rate which is a better fit by an inverse U-shaped curvature or a more positive slope using a linear model. This group difference is likely to reflect alterations in neural pruning associated with this age range. Neuronal pruning is the process of reducing the number of connections during the first two decades of life in order to select the most efficient and optimal connections, and evidence suggests that CT is likely to reflect dendritic arborization^61,62^, Accordingly, maturational changes of CT most possibly reflect the process of dendritic pruning. It is currently understood that the number of dendritic synapses reach their peak during childhood and decrease during puberty^63^. The hypothesis of altered neural, and in particular dendritic, pruning in ASD has become widespread as it is consistent with considerable evidence from multiple lines of research indicating brain overgrowth in ASD^64,65^. It has been suggested that ASD is characterized by reduced synaptic pruning^66,67^, and by genetic alterations impacting synaptic structure, function and regulation^68,69^. Our results, consistent with previous literature, give further support that atypical neural pruning in ASD is characterized by a decreased thinning rate of cortical thickness between childhood and adolescence and accelerated thinning during late adolescence.

Given that the derivative of trajectory shapes of CT across age are constant, speculatively these rates of CT change could be estimated from one subject having several longitudinal MRI acquisitions. Using cross-sectional data, we obtained a good accuracy in classifying CT trajectories, especially using the curvature of the quadratic model and using the areas from the MMP atlas. This suggests that similar accuracy could be obtained when trying to predict if individuals have ASD based on their longitudinal CT measures. To estimate the curvature in the quadratic model is necessary at least three measurements in an experiment with longitudinal design. Our results suggest that the quadratic model is the most accurate and sensitive parameter in classifying ASD, and three longitudinal time points would be necessary. It remains to be studied, however, how sensitive the curvature is in detecting individuals with ASD based solely on longitudinal samples.

## Methods

### Participants

We analyzed structural T1-weighted MRI scans from the Autism Brain Imaging Data Exchange (ABIDE)^70,71^ repository (releases I and II), which are freely accessible to the public. Quality control was assessed by visual inspection using the quality control provided by the ABIDE and by two independent reviewers (A.S.N. and N.K.). In case of disagreement, the participants were discarded if any of the independent reviewers indicated motion or magnetic artifacts. To reduce the bias in the age distribution, we excluded participants younger than 6 years of age and older than 30 years of age as beyond this age range the number of participants drops significantly. To consider the full spectrum of ASD cohort, we did not exclude participants based on their IQ. ABIDE dataset is based on studies approved by local Internal Review Boards, carried out in accordance with relevant ethics guidelines and regulations, and informed consent was obtained from all participants or, if participants were under 18, from a parent and/or legal guardian.

Out of 2,226 participants available in the ABIDE I & II, 571 were rejected based on motion artifacts detected during the visual inspection or segmentation errors revealed at the preprocessing stage, as explained below. To properly account for center variance, centers with less than 10 participants in each group were removed, which resulted in 24 centers included in the analysis. The centers with the same parameters from ABIDE I and II were combined. In total, our final sample included 674 participants with ASD (females = 94, mean 14.5 ± 5.18 years) and 686 TD participants (females = 134, mean 14.9 ± 5.49 years). Age between ASD/TD, the proportion of male/female in each center between groups or the proportion of ASD/TD participants per center did not differ significantly. Information on the participants can be found in Table 3. Four hundred and four ASD participants had information on ADOS-Generic scores (communication, social and stereotyped behavior) and were included in the analysis of associations between ADOS and age-related trajectory shapes in CT.

**Table 3:** Description of the number of participants per center, number of females per center, age mean and standard deviation.

Centers	N		Females		Mean age		STD age	
	ASD	TD	ASD	TD	ASD	TD	ASD	TD
CALTECH	14	12	3	4	22.1	22.6	3.1	3.2
CMU	10	10	1	3	23.5	24.5	3.6	3.5
KKI	58	61	16	16	10.3	10.3	1.5	1.2
LEUVEN	24	25	2	2	17.6	18.1	4.2	4.8
MAX MUN	13	13	1	0	18.3	22.8	8.1	6.7
NYU	109	116	12	23	12.5	13.8	5.8	5.4
OHSU	30	30	4	6	11.2	10.6	2.1	1.7
OLIN	16	14	3	2	16.8	16.9	3.4	3.6
PITT	24	23	4	4	16.5	17.5	4.7	4.6
SDSU	37	41	7	7	13.8	13.7	3.1	2.5
STANDFORD	15	17	4	4	10.1	9.9	1.6	1.6
TRINITY	40	45	0	0	15.9	16.5	3.4	3.5
USM	37	33	2	3	12.9	12.1	2.4	2.5
YALE	24	26	7	8	13.5	14.6	2.6	4.0
UCLA	60	53	7	11	19.3	20.0	5.2	6.1
UM	42	48	5	8	13.0	12.9	3.0	2.7
BNI	11	10	0	0	21.1	20.8	2.2	2.6
EMC	14	14	2	2	8.5	8.1	1.3	1.1
ETH	10	10	0	0	21.1	24.0	3.5	4.0
GU	31	31	4	10	11.1	11.1	1.4	1.6
IP	18	17	5	10	15.3	18.8	4.9	6.4
IU	15	17	2	5	21.5	21.9	3.5	2.0
ONRC	10	9	1	2	22.2	22.7	3.6	3.4
UCD	12	11	2	4	15.3	15.1	2.0	1.4
Total	674	686	94	134	14.5	14.9	5.2	5.5

Full names of the centers available at: http://fcon_1000.projects.nitrc.org/indi/abide/.

Our analysis was conducted on data from all the included centers combined, after correcting for batch effects (variability across centers), and, to validate the results excluding the possibility of being driven by batch effects, on a single center using the New York University (NYU) repository which has the largest sample size in the ABIDE dataset.

### Preprocessing, CT calculation and regions of interest

MRI data were segmented using FreeSurfer v5.1^72^. Cortical thickness (CT) was computed as the distance between the white and the pial surfaces for each vertex. CT values were averaged at three levels of coarseness, using three brain parcellations: an Hemispheric Parcellation (HP) with two ROIs, the Desikan-Killiany^32^ FreeSurfer Anatomical Parcellation (FSAP) atlas with 68 ROIs, and the MultiModal Parcellation (MMP) atlas with 360 ROIs which is defined by sharp changes in cortical architecture, function, connectivity, and/or topograph^33^.

To assess the presence of segmentation errors in reconstructing the cortical mantle from which CT was estimated, we conducted a second quality check. For every center separately, for each parcellation area, we performed a z-score transformation across subjects, resulting in the z-score distribution with zero mean and unit variance for each center (these normalization z-scores should not be confused with z-scores from PLS analysis). Participants with a z-score with a magnitude bigger than 3 in any area of the parcellations were considered outliers and excluded from further analysis, in total, 120 participants were discarded.

### Inter-center variability removal

Previous studies have reported variability in MRI-based measures across centers in the ABIDE repository^73^. To correct for center differences, at each area of the parcellations, linear, quadratic and cubic models were fitted to estimate the variance explained by centers while preserving the variance explained by group, ages and group x ages interactions. Specifically, the following equations were applied:

1 $$\begin{aligned} & {\text{Linear }}:{\text{CT}}\left( {{\text{Age}}} \right) \sim Intercept + {\varvec{\beta}}_{0} Center + \beta_{1 } Age + \beta_{2 } Group + \beta_{3 } \left( {Age \times group} \right) \\ & {\text{Quadratic }}:{\text{CT}}\left( {{\text{Age}}} \right) \sim Intercept + {\varvec{\beta}}_{0} Center + \beta_{1 } Age + \beta_{2 } Age^{2} + \beta_{3 } Group + \beta_{4 } \left( {Age \times group} \right) + \beta_{5 } \left( {Age^{2} \times group} \right) \\ & {\text{Cubic}}:{\text{CT}}\left( {{\text{Age}}} \right) \sim Intercept + {\varvec{\beta}}_{0} Center + \beta_{1 } Age + \beta_{2 } Age^{2} + \beta_{3 } Age^{3} + \beta_{4 } Group + \beta_{5 } \left( {Age \times group} \right) + \beta_{6 } \left( {Age^{2} \times group} \right) + \beta_{7 } \left( {Age^{3} \times group} \right) \\ \end{aligned}$$

where $Center$ represents a set of dummy variables coding a specific center (all except one to avoid multi-collinearity as we included Intercept in our models) and the ${{\varvec{\beta}}}_{0}$ coefficients in each of the three models explain the center variance for a given area. The variance explained by ${{\varvec{\beta}}}_{0}Center$ was removed for each ROI for all the three parcellations. After this procedure, CT values across centers were combined for further analyses.

### Modeling developmental trajectories of cortical thickness

Following the models from Eq. (1), we computed the slope from the linear model as the ${\beta }_{1}$ coefficient, the curvature from the quadratic model as the ${\beta }_{2}$ coefficient, and the aberrancy from the cubic model as the ${\beta }_{3}$ coefficient. These coefficients can be positive or negative based on particular changes in CT. The slope indicates a constant rate of cortical change across age, the curvature an acceleration in the rate of change and the aberrancy changes in the rate of acceleration. These geometrical shapes of the model are reflected by a line in the slope, by an upward or down-ward vertex in the curvature and by a double vertex at opposite sides of the trajectory in the aberrancy. A negative slope is inclined downwards, a negative curvature has a concave shape with an upward vertex and a negative aberrancy has a downward vertex on the left and upwards on the right side assuming the other coefficients as negative.

To capture trajectory shapes in each experimental group, we fitted the three polynomial models, separately for each group, parcellation, and brain area , using the CT data corrected for multi-center variability. The models were fitted using MATLAB function *fitglm.* Given that models were fitted separately for each group, the group and group interaction terms were removed from the models in Eq. (1), in addition to the ${{\varvec{\beta}}}_{0}$ term. Figure 1, Step 8 illustrates this analysis in the workflow.

First, these models were fitted on the entire group sample to test the goodness of fit using a deviance test of the generalized model. The analysis of deviance (as implemented in MATLAB Curve Fitting Toolbox™) measures deviance between the tested model and a constant (or null) model with the maximum number of possible parameters, and a chi-squared statistical test is used to obtain the p-value indicating if the tested model differs from the null model. Brain areas where a model did not significantly fit after FDR correction^74^ with a q = 0.05 were discarded from further analyses. In either group, if a model in a given parcellation area did not significantly fit, the area was discarded for subsequent analyses. Then, the models were fit on each subsample (or subgroup) to capture the within group variability in trajectory shapes (explained in the section below).

Given that the curvature in the quadratic model reflects acceleration, we measured the “turning point” where there is an inversion of the thinning rate, i.e. the point where decreased thinning turns into increased thinning. Negative curvatures from the ASD subgroups were selected and a straight line was traced between the CT at 6 and at 30 years old. The turning point was defined as the time point in the quadratic trajectory that was furthest from the straight line and results across trajectory areas were averaged.

### Subsampling analysis

We used subsampling to generate a distribution of the shape parameters characterizing the age-related trajectories in CT, separately for each group. This method was adapted from Vakorin et al., and Kozhemiako et al., where it was used to statistically assess group differences in the trajectory shape captured by the highest order coefficient of a model between ASD and TD populations. In our study, this subsampling method was applied to statistically assess if trajectory shapes in CT constant across age (slope, curvature or aberrancy) are atypical in the ASD group.

For each sub-sample, we randomly selected 70 participants from each group without replacement. This procedure was repeated 100,000 times. For each sub-sample, we computed the entropy of age distribution, and selected 80 sub-samples with the highest entropy for each group. The rational for this is to select sub-samples with the most uniform distributions in age (the entropy for the uniform distribution is largest). The number of repeated participants across subgroups or the entropy (age distribution) between ASD and TD groups were not significantly different.

Once the final 80 sub-sample were selected, the three models were fitted, separately for each parcellation and group, for each ROI which passed the deviance test for the goodness of fit. The coefficients representing the slope (from the linear model), curvature (from the quadratic model), and aberrancy (from the cubic model) were estimated. Thus, for each atlas (parcellation) and group, we had a matrix of shape parameters: 80 sub-samples by the number of ROIs times three (slope, curvature, and aberrancy). These data were further used for analysis of group differences with a multi-variate analysis.

### Multivariate statistical analysis

To assess group differences in the slope, curvature, and aberrancy of the developmental trajectories in CT, as well as to test for their potential associations with ASD symptomatology, we applied Partial Least Squares (PLS) analysis using the PLS toolbox (available at: www.rotman-baycrest.on.ca/source/Pls.zip) . PLS is a multivariate statistical approach that apply singular value decomposition to extract Latent Variables (LVs). Each LV is represented by a scalar (related to explained variance), a vector representing a group contrast or overall correlation, and a vector of saliences representing the contribution of each ROI to the group contrast or overall correlations between ‘imaging’ and ‘behavioral’ data^75^. In this study, two types of PLS were used: mean-centered and behavioral PLS^76,77^. The former is suitable for finding data-driven overall contrasts between groups (or conditions). For two groups, mean-centered PLS is equivalent to testing a group contrast, which was defined in our case as the ASD > TD hypothesis. In turn, the behavioral PLS explores associations between two matrices: ’imaging’ data (CT estimates, or the slope, curvature, and aberrancy) and ‘behavioral’ data (ADOS scores, in our case). Previously, behavioral PLS has been used to find behavioral associations with BOLD MRI^76,78,^ and neuromagnetic brain activity^75,79–81^. Both approaches perform a permutation test by resampling without replacement all subjects’ assignment across groups, and a unique p-value for all the features is obtained by counting the number of times the corresponding singular value for each permutation was higher than the original one. Then, bootstrapping is performed by resampling participants with replacement while keeping the group assignment fixed. The original saliences were divided by the bootstrap standard error to obtain a bootstrap ratio associated with each feature (ROI) indicating the reliability of the feature’s contribution to the identified contrast (or overall correlation). These bootstrap ratio values are similar to z-scores, and in our study, we used these terms interchangeably. Thus, each feature (in our case a coefficient expressing a trajectory shape for a given area in a given parcellation) is associated with a z-score which indicates the reliability of the contribution of the given brain area to the group contrast or overall correlation.

### Analysis of group differences in trajectory shapes in CT

Three independent mean-centered PLS analyses were conducted separately for the HP, FSAP and MMP to explore differences in the trajectory shapes between ASD and TD groups. For each PLS analysis, the input was a matrix of the shape estimates: 80 sub-samples within two groups by the number of ROIs times three (three parameters). Permutations and bootstrapping were performed 10,000 times to obtain statistically reliable results. Assuming the contrast ASD > TD, positive z-scores are associated with brain regions wherein the trajectory shape in CT is increased in the ASD group. Correspondingly, negative z-scores indicate brain areas with a decreased trajectory shape value in ASD. The z-scores were separated for the slope, curvature, and aberrancy and their distributions were found to be skewed, indicating that there is a predominant increased or decreased effect representing the group differences. To visualize the results, the threshold was set as the highest value opposite of the skewness.

### Center-specific analysis of group differences in trajectory shapes in CT

The same subsampling analysis of group differences was repeated for one center (NYU). The number of participants per sub-sample was reduced to 40, and the number of subgroups to 50. We aimed to further support our results, by explicitly avoiding possible confounding effects of the inter-center variability.

### Associations between ADOS scores and trajectory shapes in CT

To explore the relationship between ASD symptomatology and trajectory shapes of CT maturation, behavioral PLS correlated the slope, curvature, and aberrancy of the corresponding polynomial models of age-related changes in CT, which was computed for sub-samples of participants with the subgroup mean ADOS-Generic scores (communication, social and stereotyped behavior subscales). For each of the three model coefficients (linear slope, curvature and aberrancy) we performed two PLS analyses, separately for the FSAP and MMP parcellations)(six PLS analyses in total), and the p-values obtained for each model were Bonferroni corrected.

The behavioral PLS analysis renders a latent variable (LV) design vector indicating the contribution of the ADOS sub-score (communication, social and stereotyped behavior), a correlation for each ADOS sub-score, a single p-value for the overall-correlation, and z-scores expressing the stability of the feature in expressing the correlation. To make contributions of individual participants more pronounced, the number of participants per subgroup was decreased to 20, and to increase the combination of subjects, the number of subgroups was increased to 400.

### ASD classification of CT developmental trajectories

To assess which model parameter can better predict ASD versus TD status, a linear kernel support vector machine (SVM, using MATLAB 2018b *fitcsvm* function with default parameters) was trained to classify trajectory shapes from ASD and TD subgroups using the FSAP or MMP areas.

First, experimental groups from the full dataset were split into two equal training and testing sets. To have a similar age, for each experimental group, participants were separated within two years age bins, and the participants in each bin were randomly split in two. Once the training and testing sets were created, for each set, the subsampling analysis was applied (the CT values from the FSAP and MMP areas using 80 subgroups were fitted using the three models and the three coefficients were obtained). Then, an SVM was trained using the training set to classify one model coefficient from the FSAP or MMP, and the testing set was used to measure the accuracy in classifying ASD and TD using the slope, curvature or aberrancy. The accuracy represents the percentage of correctly classified ASD and TD trajectories together whereas the sensitivity and specificity are the percentages of correctly classified ASD and TD trajectories, respectively. This procedure was repeated 500 times (500 cross-validations) to obtain a distribution of accuracy, sensitivity and specificity, and a t-test was used to test for differences between models of a parcellation. In addition, we analyzed if accuracy, sensitivity or specificity across cross-validations was correlated with the number of females in the subgroup or the mean ADOS for the ASD group.

## Supplementary information

Supplementary file1 (DOCX 8227 kb)

## Data Availability

Data is available from the ABIDE repository. The codes for the analyses are publicly available at https://github.com/AdoNunes/CT_trajectory_features_ASD_2019.

## Abbreviations

ASD: Autism spectrum disorder
TD: Typically developing
CT: Cortical thickness
ADOS: Autism diagnostic observation schedule
PLS: Partial least squares
