# Supplementary material for: Atypical age-related changes in cortical thickness in autism spectrum disorder
Source: Sci Rep. 2020 Jul 6;10:11067. doi: 10.1038/s41598-020-67507-3 (PMC7338512; doi:10.1038/s41598-020-67507-3)
Supplement: Supplementary file 1 — Supplementary file1 (DOCX 8227 kb) [file 41598_2020_67507_MOESM1_ESM.docx]

**Atypical age-related changes in cortical thickness in Autism Spectrum Disorder**

Authors:

Adonay S Nunes^1^ǂ, Vasily A Vakorin^1,2^, Nataliia Kozhemiako^1^, Nicholas Peatfield^1^, Urs Ribary^2,3,4,5^, Sam M Doesburg^1,2^

Affiliations:

1 Department of Biomedical Physiology and Kinesiology, Simon Fraser University, Canada

2 Behavioral & Cognitive Neuroscience Institute, Simon Fraser University, Canada

3 Department Pediatrics and Psychiatry, University of British Columbia, Canada

4 B.C. Children's Hospital Research Institute, Canada

5 Department Psychology, Simon Fraser University, Canada

ǂ Correspondence:

[anunessa@sfu.ca](mailto:anunessa@sfu.ca)

Biomedical Physiology Department,

Simon Fraser University,

8888 University Dr,

Burnaby, BC V5A 1S6,

Canada


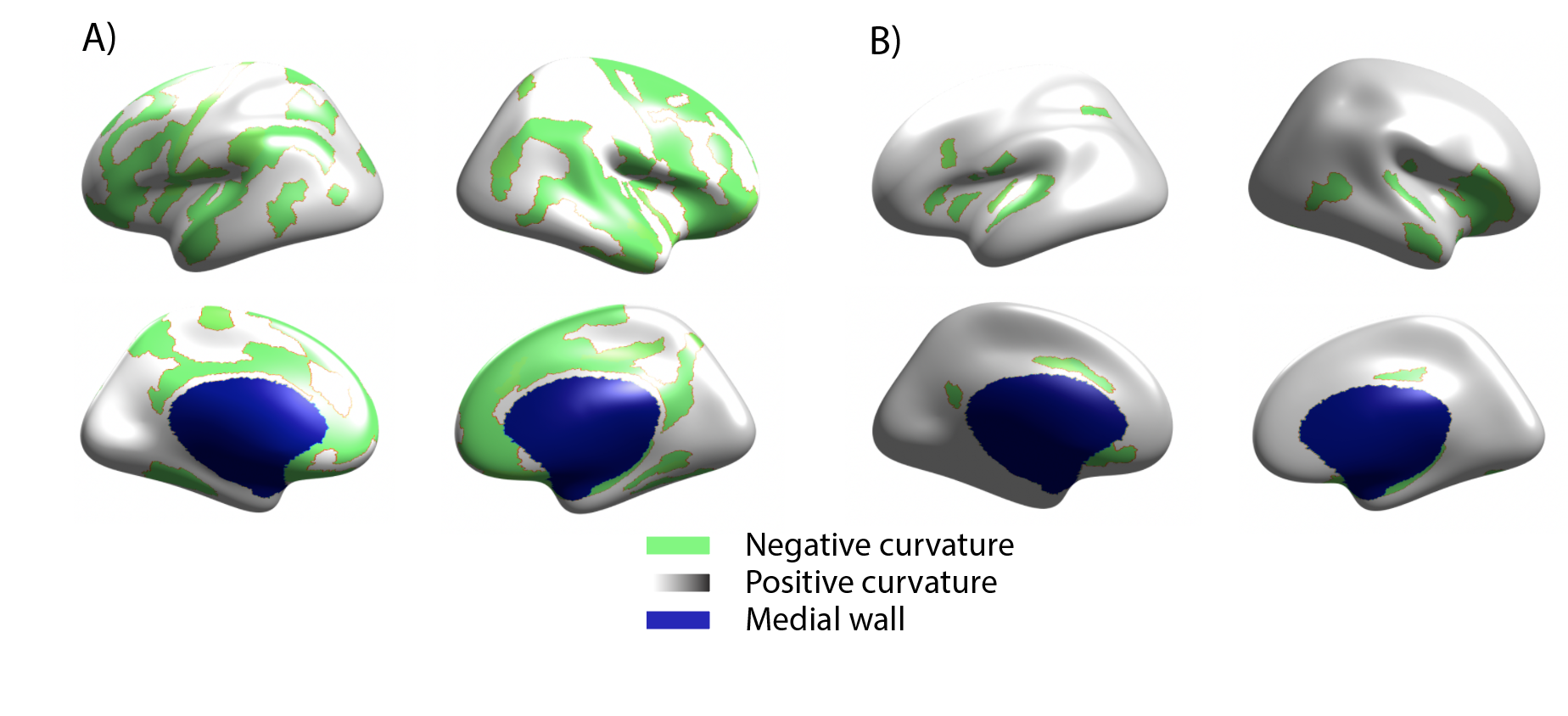


***Supplementary Figure 1.*** Areas with negative subgroup-mean curvature values in A) ASD and B) TD. Green areas represent negative curvatures whereas white are positive. The spatial distribution of negative curvature areas in the ASD greatly overlaps the areas with the largest z-scores in the PLS group analysis.


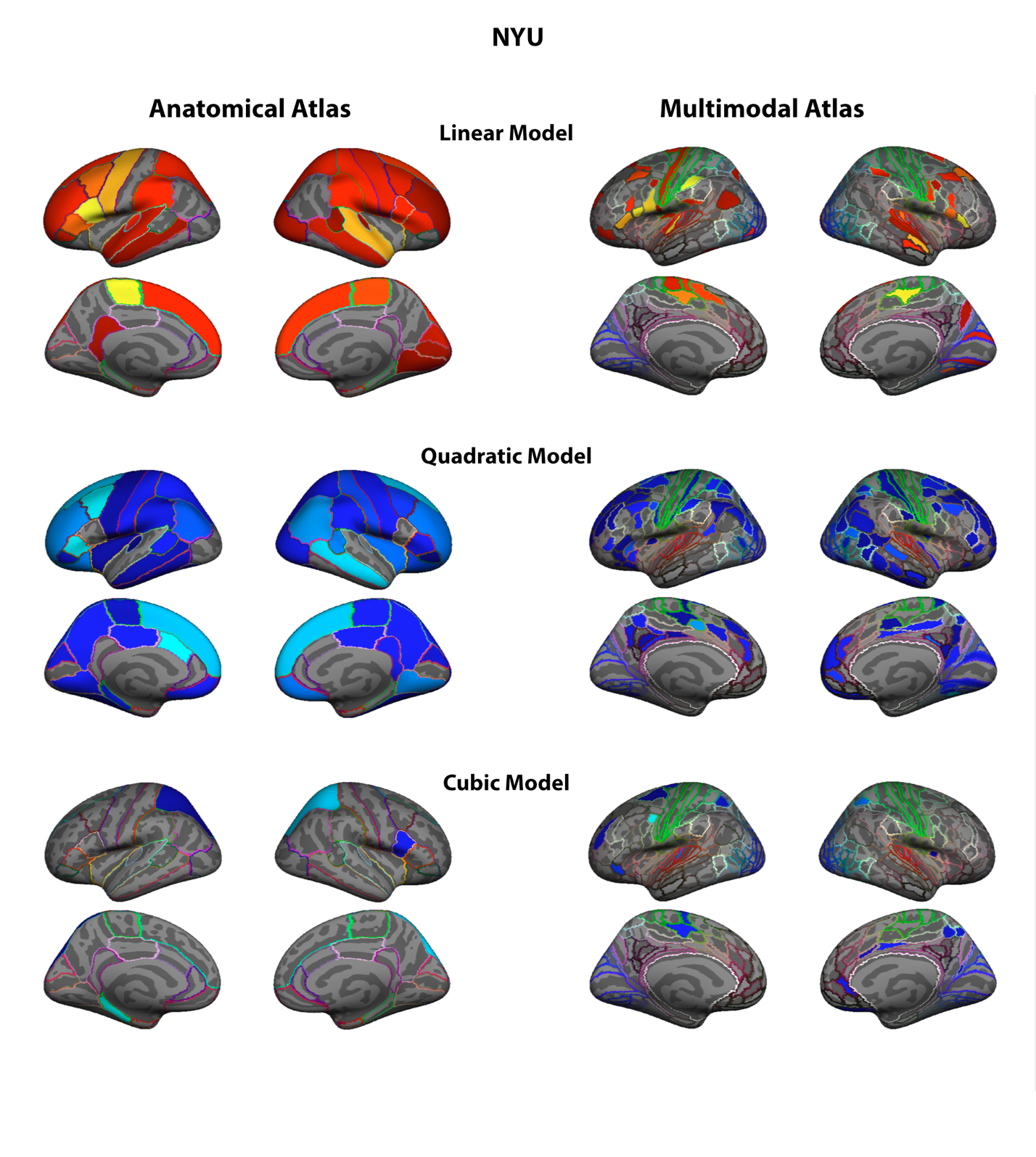


***Supplementary Figure 2. Group differences in rate of changes in CT maturation using the NYU center.*** *For the group contrast (ASD>TD) the effect found in the linear coefficient is positive, whereas in the quadratic and cubic is negative. The spatial distribution of the highest z-scores in the linear model are very similar to the quadratic, and both represent a decreased cortical thinning during childhood to adolescence maturation but the quadratic also captures accelerated thinning in the later age period. On the left are the results from the anatomically defined FreeSurfer atlas and on the right the Multi-Modal Parceled atlas.* *Outlined colors in each cortical area represent the original atlas annotation color of the area.*

**
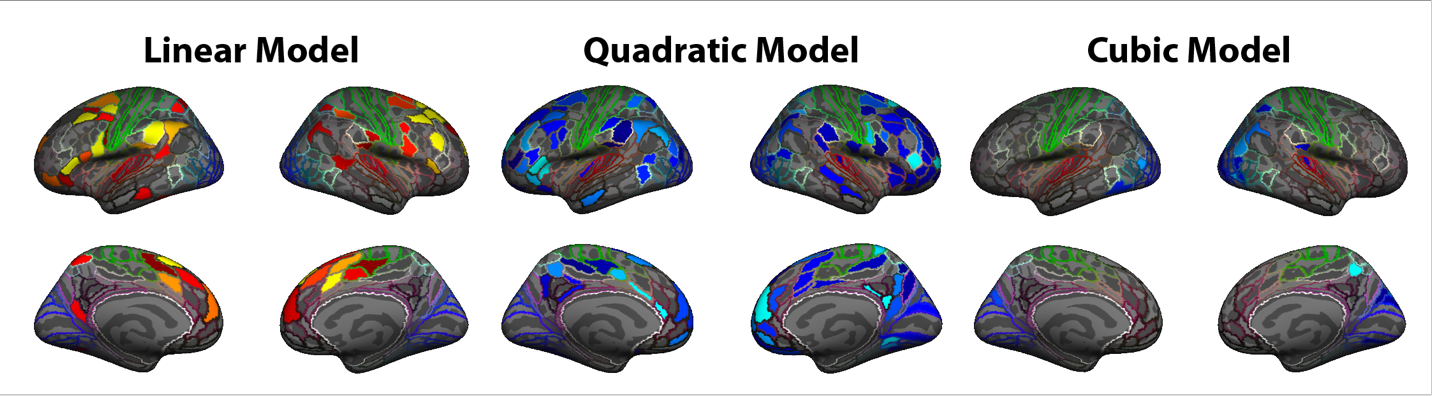
**

***Supplementary Figure 3. Group differences in developmental trajectory shapes accounting for group differences.*** *The same PLS analysis as illustrated in Fig. 4 was applied but besides removing the variance explained by center variability also the variance explained by sex was also removed. The results are very similar to the original analysis illustrated in Fig. 4 indicating that sex differences were not driving the group results.*
